# Accuracy of brain natriuretic peptide and N-terminal brain natriuretic peptide for detecting paediatric pulmonary hypertension: a systematic review and meta-analysis

**DOI:** 10.1080/07853890.2024.2352603

**Published:** 2024-05-16

**Authors:** Ruixi Zhou, Yupeng Lei, Long Ge, Qian Mao, Liuping Yang, Xia Qiu

**Affiliations:** a Department of Pediatrics, West China Second University Hospital, Key Laboratory of Obstetric and Gynecologic and Pediatric Diseases and Birth Defects of Ministry of Education, Sichuan University, Chengdu, China; b Evidence-Based Social Science Research Center, School of Public Health, Lanzhou University, Lanzhou, China; c Department of Social Medicine and Health Management, School of Public Health, Lanzhou University, Lanzhou, China

**Keywords:** Pulmonary hypertension, paediatric, brain natriuretic peptide, N-terminal brain natriuretic peptide, sensitivity, specificity

## Abstract

**Objective:**

Pulmonary hypertension (PH) is a life-threatening disease, especially in paediatric population. Symptoms of paediatric PH are non-specific. Accurate detection of paediatric PH is helpful for early treatment and mortality reduction. Therefore, we assessed the overall performance of brain natriuretic peptide (BNP) and N-terminal brain natriuretic peptide (NT-proBNP) for diagnosing PH in paediatric population.

**Methods:**

PubMed, Web of Science, Cochrane Library and Embase databases were screened since their respective inceptions until August 2023. A bivariate random model and a hierarchical summary receiver operating characteristic model were used together to evaluate and summarize the overall performance of BNP and NT-proBNP for diagnosing paediatric PH.

**Results:**

Eighteen studies using BNP/NT-proBNP were assessed, comprising 1127 samples. The pooled sensitivity, specificity, positive likelihood ratio (PLR), negative likelihood ratio (NLR), diagnostic odds ratio (DOR) and area under the curve (AUROC) of BNP/NT-proBNP were separately as 0.81, 0.87, 6.33, 0.21, 29.50 and 0.91, suggesting a good diagnostic performance of BNP/NT-proBNP for detecting PH in paediatric population. For BNP, the pooled sensitivity, specificity, PLR, NLR, DOR and AUROC were 0.83, 0.89, 7.76, 0.19, 40.90 and 0.93, indicating the diagnostic accuracy of BNP for paediatric PH patients was good. For NT-proBNP, the pooled sensitivity, specificity, PLR, NLR, DOR and AUROC were 0.81, 0.86, 5.59, 0.22, 24.96 and 0.90, showing that NT-proBNP could provide a good value for detecting paediatric PH.

**Conclusions:**

Both BNP and NT-proBNP are good markers for differentiating paediatric PH patients from non-PH individuals.

## KEY MESSAGES

Accurate detection of paediatric PH is helpful for early treatment and mortality reduction. This study shows that both BNP and NT-proBNP are good markers for detecting paediatric PH. In clinical practice, we recommend that BNP and NT-proBNP are auxiliary biomarkers in diagnosing paediatric PH.

## Introduction

Pulmonary hypertension (PH) is a life-threatening disease with a complex aetiology, especially in paediatric population [1,2]. The common causes distributed for paediatric PH are congenital heart disease (CHD-PH), idiopathic PH and pulmonary disorders, like bronchopulmonary dysplasia (BPD-PH), neonatal persistent pulmonary hypertension (PPHN), etc [3–5]. It was reported that a prevalence of 20 to 40 cases per million paediatric individuals had PH in Europe [6]. Besides, a national epidemiological study by van Loon et al. showed an estimated incidence of CHD-PH was 2.2 cases per million children annually [7]. Symptoms of paediatric PH are non-specific and delayed diagnosis and treatment typically caused heart failure and death.

In the absence of typical clinical manifestations, making the correct diagnosis among children with PH is significant and challenging. Historically, the diagnostic standard of paediatric PH is a mean pulmonary artery pressure (mPAP) greater than 25 mmHg at rest [8,9]. From 2018, PH was defined in children as a mPAP above 20 mmHg, recommended by the Pediatric Task Force of the 6th World Symposium on Pulmonary Hypertension [6,10]. Cardiac catheterization is usually used to accurately measure mPAP; however, it is an invasive procedure which requires general anaesthesia or conscious sedation [6]. Paediatric PH patients are a high-risk population for diagnostic cardiac catheterization. It was reported that the risk of catastrophic adverse events after catheterization in children was 1.4%, in which the mortality risk before discharged was 5.2% [11]. Doppler echocardiography is a non-invasive method for PH diagnosis, but well-experienced experts are needed to perform and explain the images [2,12]. Meanwhile, it has great subjectivity in the shortage of adequate signals. In the light of the limitations of routine measures as stated above, additional diagnostic methods for children with PH are the extremely urgent request.

Biochemical markers, which could be quickly detected from body fluids, have provided potential tools for the diagnosis in the field of children with PH [12]. Brain natriuretic peptide (BNP) and its N-terminal cleavage protein, named N-terminal natriuretic peptide (NT-proBNP), are produced by heart muscle cells and secreted into the cardiovascular system, in response to ventricular wall stress due to pressure overload [13]. The endocrine effects on cardiovascular system of BNP and NT-proBNP are mainly mediated *via* guanylate cyclase-A coupled receptors [14]. Besides, BNP has a shorter half-life than NT-proBNP (18 min vs. 118 min). Recently, many studies have mentioned that BNP and NT-proBNP could provide a potential additive value for detecting paediatric PH [2,12,15–30]. However, until now, the diagnostic accuracy of BNP and NT-proBNP among pediatric population with PH remains various [2,12,15–30]. Therefore, in this study, we conducted a systematic review and diagnostic meta-analysis to evaluate the overall performance of BNP and NT-proBNP in detection of paediatric PH.

## Materials and methods

### Search strategy

The 2018 and 2020 guidelines from the Preferred Reporting Items for Systematic Reviews and Meta-Analyses Diagnostic Test Accuracy (PRISMA-DTA) statement, explanation and elaboration published by McInnes et al. were followed (Supplementary checklist) [31,32]. Our team registered this protocol on PROSPERO (CRD42023388033). The publications were screened from the electronic databases, including PubMed, Web of Science, Cochrane Library and Embase, since their respective inceptions until August 2023. We used the search terms as follows: (pulmonary hypertension OR pulmonary arterial hypertension OR PH) AND (brain natriuretic peptide OR B type natriuretic peptide OR BNP OR N-terminal brain natriuretic peptide OR pro-brain natriuretic peptide OR amino terminal pro brain natriuretic peptide OR N-terminal pro-B-type natriuretic peptide OR amino terminal pro-B-type natriuretic peptide OR N-terminal pro brain natriuretic peptide OR proBNP OR NT-proBNP) AND (child OR children OR pediatric OR infant OR newborn OR neonate OR premature). Additionally, the potential studies were searched from the reference lists of identified articles and relevant reviews. Our study didn’t need an approval of the institutional ethics Committee, as well.

### Selection of studies

Two authors (XQ, RZ; in pairs) screened titles, abstracts, and full texts of the search results independently, and any discrepancy were resolved by discussion. The detailed selection criteria of studies reporting BNP/NT-proBNP for diagnosing PH in paediatric population was as follows: (1) participants included PH patients and non-PH controls ranging in age from 0 to 18 years old; (2) index test was BNP and/or NT-proBNP; (3) diagnostic standards for children with PH were the mPAP over 20 mmHg or 25 mmHg, and/or right ventricular pressure >1⁄2 systemic pressure, and/or interventricular septal flattening, and/or right ventricular hypertrophy, which measured by cardiac catheterization and/or echocardiography [1,33]; (4) outcomes were sensitivity and specificity of BNP and/or NT-proBNP, as well as other numerical data which could computed both these measures; (5) study designs included randomized controlled trials and prospectively/retrospectively cohort trials and cross-sectional studies. The optimal cut-off was chosen as two or more cut-offs were reported in the same original study.

We imposed no restrictions on study language. Animal or in-vitro experiments, case reports or case series, narrative reviews, guidelines, recommendations, patents, systematic review/meta-analysis and studies without a control group were excluded.

### Data extraction and management

Two authors (XQ, RZ; in pairs) independently extracted a standard set of data from the eligible articles, and deviations were resolved by consensus. A predefined data extraction content was as follows: the first author, year of publication, country, study design, disease type (CHD-PH, BPD-PH, PPHN, etc.), number of participants (PH patients/non-PH controls), index test type (BNP/NT-proBNP), the cut-off of index test and the diagnostic reference standard of paediatric PH. Additionally, sensitivity, specificity, true positive (TP), false positive (FP), false negative (FN), and true negative (TN) data of BNP/NT-proBNP for diagnosing paediatric PH were extracted.

### Methodological quality assessment

The quality assessment of diagnostic accuracy studies-2 (QUADAS-2) checklist was utilized to summarize the quality of included studies, by two authors (XQ, RZ; in pairs) [34]. Furthermore, we used a RevMan software (version 5.3; Cochrane, London, UK) to generate the figure of methodological quality assessment.

### Statistical analysis

To generate the pooled estimates of sensitivity, specificity, positive likelihood ratio (PLR), negative likelihood ratio (NLR), and diagnostic odds ratio (DOR) value for BNP/NT-proBNP, we applied bivariate random meta-analysis methods [35]. Besides, the area under the summary receiver operating characteristic curve (AUROC) was calculated to judge the diagnostic accuracy of BNP and NT-proBNP in detecting PH in paediatric population. The diagnostic effect is more reliable as the AUROC value is greater [36]. An AUROC between 0.75 and 0.93 or exceeding 0.93 indicated that the diagnostic performance of BNP/NT-proBNP was good or excellent, respectively [37]. Meanwhile, we used a hierarchical summary receiver operating characteristic (HSROC) curve to summarize the overall diagnostic performance of BNP/NT-proBNP [38].

We predefined heterogeneity as an *I^2^* statistic over 75% and calculated it where there were more than five studies [39]. Where heterogeneity was shown, meta-regression and subgroup analyses were utilized to explore the potential causes of heterogeneity. Subgroups were prespecified covariates, including different study design (cohort or not), disease type (BDP-PH or not) and cut-offs (≥100 pg/ml or not). Additionally, Deeks’ funnel plot was used to evaluate the possible existence of publication bias, in which an evidence of publication bias was found with *p* < 0.10 [40]. The statistical software Stata (version 18.0; StataCorp, College Station, TX, USA) was used to pool and analyse the diagnostic data.

## Results

### Characteristics of the included articles

**Figure 1.**
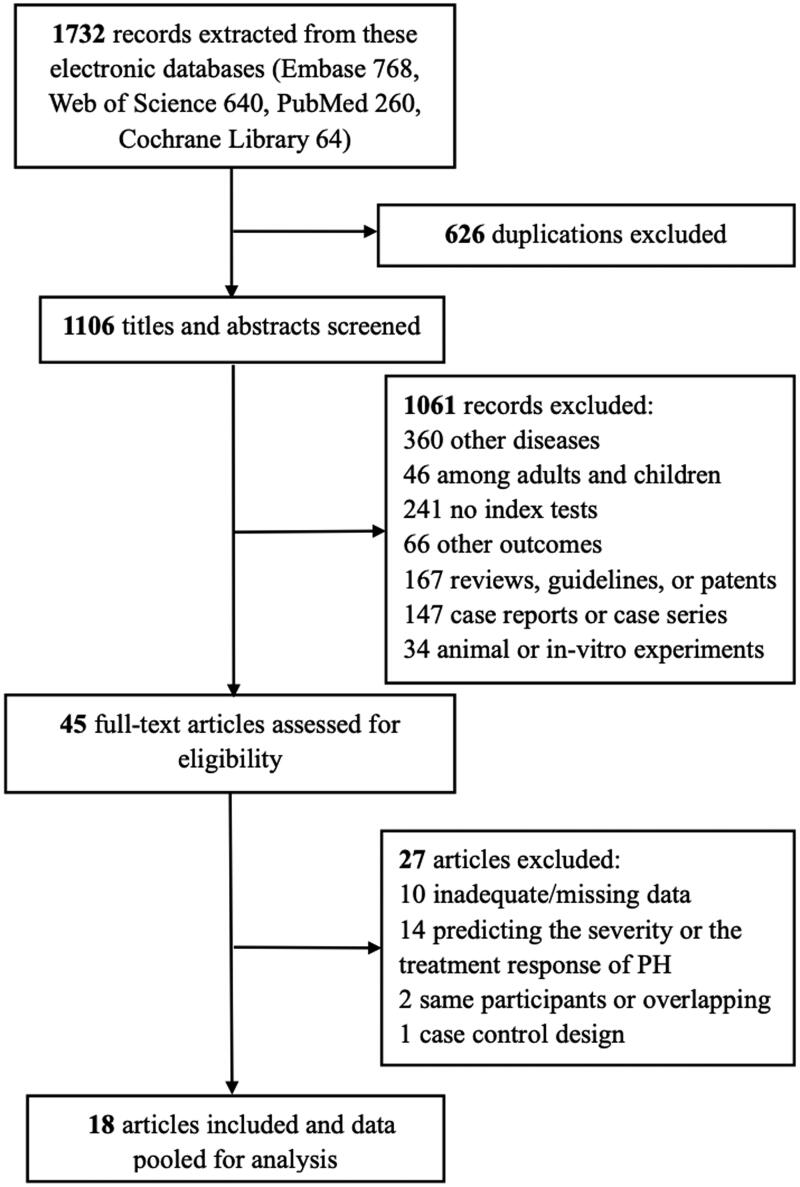
The selection process of included studies.

We extracted 1732 records across electronic databases, of which 626 duplications were ruled out. After 1106 titles and abstracts were reviewed, we excluded 1061 records, of which 360 records were not eligible with other diseases (heart failure, patent ductus arteriosus, congenital diaphragmatic hernia, systemic sclerosis, etc.) and 46 records were not focused on the specific population (adults only, adults and children); 241 records were other index tests (troponin T, lipocalin 2, growth differentiation factor-15, circular RNA, etc.) and 66 records were other outcomes; 167 records were non-eligible types of articles (reviews, guidelines, patents, systematic review/meta-analysis, etc.); 147 records were case reports or case series; and 34 records were of animal or in-vitro experiments. Then, 45 full-text articles were assessed for eligibility (Figure 1). Ultimately, 18 articles were included for characteristics, and data were pooled for meta-analysis assessments [2,12,15–30].

Detailed characteristics of eighteen publications are presented in Table 1. Overall, 498 diagnosed PH patients and 629 non-PH individuals entered this study, ranging in age from 0 to 18 years old. Nine (50%) articles were reported from USA [5,18–21,23,27–29], and four (22%) articles originated from China [2,17,25,26]. 50% and 50% of studies were of prospective/retrospective cohort studies and cross-sectional studies between 2003 and 2023, respectively. The patients all had PH, of which six publications (33%) focused on BPD-PH patients [19–21,23,27,28], eight publications (44%) paid attention to CHD-PH populations [2,5,15,17,24–26,30], and two publications (11%) concentrated on PPHN newborns [16,29]. As to reference standard, seven studies (39%) used cardiac catheterization to detect pulmonary artery or right ventricular pressure, while 11 studies (61%) utilized echocardiography to calculate them. Seven studies (39%) reported BNP as the index test and eleven studies (61%) studies reported NT-proBNP. The cut-offs were ranging from 20 to 550 pg/mL for BNP and 112 to 2760 pg/mL for NT-proBNP. Furthermore, diagnostic accuracy estimates of BNP for detecting PH in paediatric population are shown in Supplementary table 1.

**Table 1.** Characteristics of the included articles.

Author	Year	Country	Study design	Disease type	PH/non-PH participants (N)	Index test		Reference standard
						Type	Cut-off (pg/ml)	
Jin N [2]	2023	China	Cross-sectional	CHD-PH	65/92	NT-proBNP	492	Cardiac catheterization
Nova R [15]	2022	Indonesia	Cross-sectional	CHD-PH	33/42	NT-proBNP	554	Echocardiography
Tufekci S [16]	2022	Turkey	Cross-sectional	PPHN	33/50	NT-proBNP	2760	Echocardiography
Zhang H [17]	2021	China	Prospective cohort	CHD-PH	54/25	NT-proBNP	521	Cardiac catheterization
Dasgupta S [5]	2021	USA	Prospective cohort	CHD-PH	10/21	NT-proBNP	408	Cardiac catheterization
Griffiths M [18]	2020	USA	Prospective cohort	PH	26/21	NT-proBNP	262	Echocardiography
Naeem B [19]	2020	USA	Cross-sectional	BPD-PH	6/20	NT-proBNP	2345	Echocardiography
Behere S [20]	2019	USA	Retrospective cohort	BPD-PH	7/21	BNP	100	Echocardiography
Avitabile CM [21]	2019	USA	Retrospective cohort	BPD-PH	68/60	BNP	130	Echocardiography
Rodriguez-Gonzalez M [22]	2019	Spain	Prospective cohort	PH	21/72	NT-proBNP	1345	Echocardiography
Dasgupta S [23]	2018	USA	Prospective cohort	BPD-PH	8/28	NT-proBNP	2329	Echocardiography
Pektaş A [24]	2017	Turkey	Retrospective cohort	CHD-PH	25/40	BNP	305	Cardiac catheterization
Li G [25]	2017	China	Cross sectional	CHD-PH	46/39	NT-proBNP	112	Cardiac catheterization
Li G [26]	2016	China	Cross sectional	CHD-PH	30/30	BNP	Un	Cardiac catheterization
Montgomery AM [27]	2016	USA	Cross-sectional	BPD-PH	5/15	NT-proBNP	1000	Echocardiography
Cuna A [28]	2013	USA	Cross-sectional	BPD-PH	16/9	BNP	117	Echocardiography
Reynolds EW [29]	2004	USA	Prospective cohort	PPHN	15/15	BNP	550	Echocardiography
Suda P [30]	2003	Japan	Cross-sectional	CHD-PH	30/29	BNP	20	Cardiac catheterization

Abbreviations: PH: pulmonary hypertension; CHD: congenital heart disease; PPHN, neonatal persistent pulmonary hypertension; BPD: bronchopulmonary dysplasia; BNP: brain natriuretic peptide; NT-proBNP: N-terminal pro-brain natriuretic peptide.

### Methodological quality of the eligible studies

**Figure 2.**
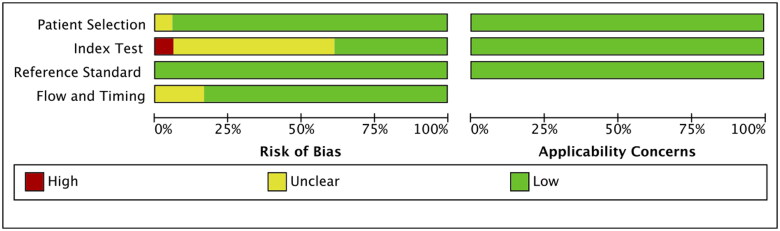
Quality for the included articles.

We found a relatively low risk of methodological quality bias for these included studies using the QUADAS-2 framework (Figure 2). For patient selection, the risk of bias was unclear for one study (6%) as consecutive selection and inclusion time of patients with PH were not described [2]. For index test, the risk of bias was unclear in most studies, because it was difficult to judge whether the index test was performed blinded; Besides, the risk of bias was assessed as high for one study as the cut-off value of BNP was not reported [26]. For reference standard, the low bias was existed as an appropriate reference standard (cardiac catheterization or echocardiography) was selected in these included studies. Flow and timing bias was unclear for three articles (17%), as investigators directly excluded the missing data of paediatric PH patients and non-PH individuals [19,20,23].

### Diagnostic accuracy of BNP and NT-proBNP

**Figure 3.**
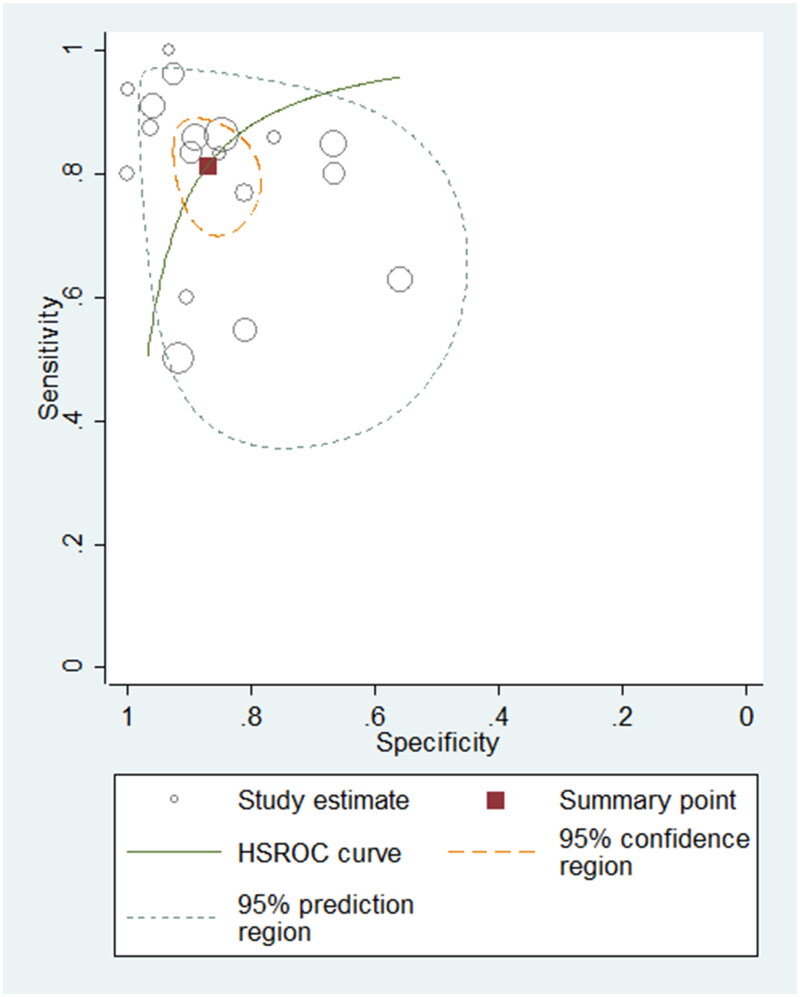
Hierarchical summary receiver operating characteristic plot to summarize diagnostic performance of BNP/NT-proBNP for detecting paediatric PH.

To differentiate PH patients from non-PH controls, eighteen studies using BNP/NT-proBNP were assessed, comprising 1127 samples. The sensitivity varied between 0.50 and 1.00 in these eligible studies, with a pooled sensitivity of 0.81 (95% confidence intervals (CI): 0.73–0.87, *I*^2^: 78.55%) (Supplementary Figure 1). The specificity ranged from 0.56 to 1.00, with a pooled specificity of 0.87 (95% CI: 0.81–0.91, *I*^2^: 72.99%) (Supplementary Figure 1). The pooled PLR, NLR and DOR were 6.33 (95% CI: 4.14–9.69), 0.21 (95% CI: 0.15–0.32) and 29.50 (95% CI: 14.40–60.45), respectively. The AUROC was 0.91 (95% CI: 0.88–0.93), indicating the diagnostic performance of BNP/NT-proBNP for detecting PH in paediatric population was good. The HSROC curve of BNP/NT-proBNP is shown in Figure 3.

### Diagnostic accuracy of BNP

**Figure 4.**
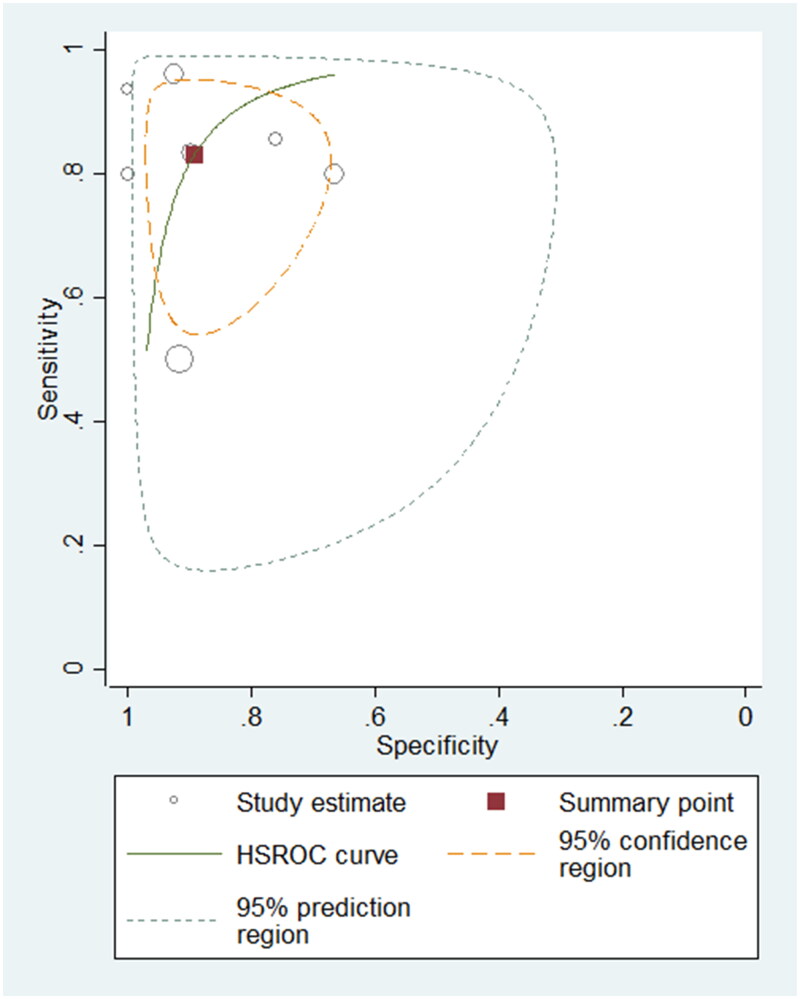
Hierarchical summary receiver operating characteristic plot to summarize diagnostic performance of BNP for detecting paediatric PH.

A total of 395 samples from seven studies using BNP were evaluated. The summary sensitivity and specificity were 0.83 (95% CI: 0.68–0.92; *I^2^*: 85.45%) and 0.89 (95% CI: 0.79–0.95; *I^2^*: 77.99%), respectively. The summary PLR, NLR and DOR were 7.76 (95% CI: 3.69–16.32), 0.19 (95% CI: 0.09–0.38) and 40.90 (95% CI: 12.53–133.45), separately. The AUROC was 0.93 (95% CI: 0.91–0.95), suggesting that the diagnostic accuracy of BNP for differentiating PH patients from non-PH controls was good. The HSROC curve of BNP is shown in Figure 4.

We observed a relatively high heterogeneity existing (*I^2^* = 85% for sensitivity). In the meta-regression and subgroup analyses, the different study design (cohort or not), disease type (BDP-PH or not) and cut-off (≥100 pg/ml or not) might not be the cause of heterogeneity (*p* = 0.53, 0.60, and 0.08). For study design, the pooled sensitivity and specificity of cohort studies were 0.79 and 0.91, respectively. In addition, the pooled sensitivity and specificity of cross-sectional studies were 0.87 and 0.85, separately. For different diseases of PH, the pooled sensitivity of BDP-PH was less than of other PH diseases (0.74 vs. 0.87); however, the pooled specificity of BDP-PH and other PH diseases was similar (0.90 and 0.89). The pooled sensitivity of cut-off ≥100 pg/ml was smaller when compared with cut-off <100 pg/ml (0.77 vs. 0.87), while the pooled specificity was opposite (0.94 vs. 0.81).

### Diagnostic accuracy of NT-proBNP

**Figure 5.**
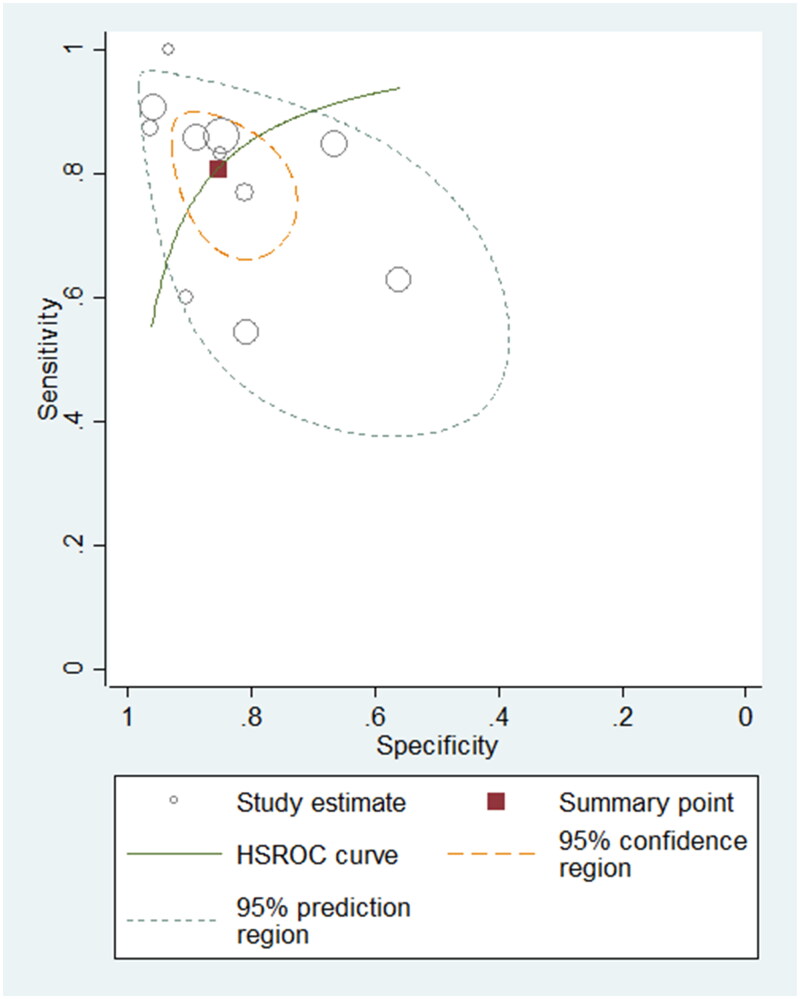
Hierarchical summary receiver operating characteristic plot to summarize diagnostic performance of NT-proBNP for detecting paediatric PH.

Eleven studies, comprising 732 samples, were included in the NT-proBNP group. The pooled sensitivity and specificity were 0.81 (95% CI: 0.72–0.88; *I^2^*: 73.13%) and 0.86 (95% CI: 0.78–0.91; *I^2^*: 73.06%), respectively. The pooled PLR and NLR were 5.59 (95% CI: 3.36–9.31) and 0.22 (95% CI: 0.14–0.35), respectively. The summary DOR was 24.96 (95% CI: 10.27–60.67). The AUROC was 0.90 (95% CI: 0.87–0.92), suggesting that the diagnostic value of NT-proBNP for detecting PH among children was good. The HSROC curve of NT-proBNP is shown in Figure 5.

A relatively high heterogeneity was observed between the included studies (*I^2^* = 73% for sensitivity). In the meta-regression and subgroup analyses, we found no statistically significant differences within the different study design (cohort or not), disease type (BDP-PH or not) and cut-off (≥100 pg/ml or not) (*p* = 0.67, 0.23, 0.35), indicating they might not be the cause of heterogeneity. The pooled sensitivity and specificity of cohort studies were smaller than of cross-sectional studies (sensitivity: 0.77 vs. 0.83, specificity: 0.85 vs.0.86). For different diseases of PH, the pooled sensitivity and specificity of BDP-PH were more than of other PH diseases (sensitivity: 0.90 vs. 0.78, specificity: 0.93 vs. 0.83). The pooled sensitivity of cut-off ≥100 pg/ml was less when compared with cut-off <100 pg/ml (0.79 vs. 0.82); however, the pooled specificity of cut-off ≥100 pg/ml was higher when compared with cut-off <100 pg/ml (0.88 vs. 0.80)

### Publication bias

Deeks’ funnel plots revealed no significant difference in BNP/NT-proBNP (*p* = 0.35), BNP (*p* = 0.29) and NT-proBNP (*p* = 0.78), separately (Supplementary Figure 2a-c). We found no evidence of publication bias in this study.

## Discussion

It is well known that BNP and NT-proBNP are recommended for the diagnosis and prognosis of heart failure [41]. In this study, the results showed that BNP/NT-proBNP displayed a good diagnostic performance for detecting paediatric PH, with an AUROC of 0.91. Besides, our results suggested that BNP demonstrated a good diagnostic accuracy for detecting paediatric PH patients, with an AUROC of 0.93. NT-proBNP also provided a good value for differentiating paediatric PH patients from non-PH individuals, with an AUROC of 0.90. Furthermore, our analysis revealed the different study design (cohort or not), disease type (BDP-PH or not) and cut-off (≥100 pg/ml or not) of both BNP and NT-proBNP were not the cause of heterogeneity.

In 2022, Zhang et al. suggested that BNP/NT-proBNP had a certain diagnostic performance for detecting PH with a moderate sensitivity (0.67) and good specificity (0.84) [42]. However, this previous diagnostic meta-analysis concentrated on systemic sclerosis-associated PH among adults. In 2015, a systematic review published by ten Kate et al. including 14 studies, showed that BNP/NT-proBNP could not be good at detecting paediatric PH, by the deficiency of absolute normal range values [43]. Meanwhile, the summary sensitivity and specificity of BNP/NT-proBNP were not reported, with a small number of participants. To date, the diagnostic accuracy of BNP and NT-proBNP for detecting paediatirc PH remained unclear. In this study, BNP/NT-proBNP had a relatively high summary sensitivity (0.81) and specificity (0.87). It also signified a relatively low rate to escape diagnosis (19%) and be misdiagnosed (13%). The AUROC was 0.95, suggesting that BNP/NT-proBNP could be good at detecting PH among children. In clinical practice, we recommend that BNP and NT-proBNP are auxiliary biomarkers for heart catheterization and/or echocardiography in differentiating paediatric PH patients from non-PH individuals.

Many studies have found that BNP has a shorter half-life and lower stability than NT-proBNP in plasma [42,44,45]. However, our study revealed a slightly higher sensitivity (0.83) and specificity (0.89) of BNP according to data from seven studies, whereas the sensitivity and specificity of NT-proBNP were 0.81 and 0.86 based on 11 studies, respectively. It showed that the diagnostic accuracy of BNP for pediatric PH was slightly superior to NT-proBNP (AUROC: 0.93 vs. 0.90). Therefore, more multi-centre and large sample studies are needed for exploration and comparison of the diagnostic value of BNP and NT-proBNP for PH among children.

Our meta-analysis had several limitations. First, misclassification bias could not be ruled out, because not all studies used cardiac catheterization to measure mPAP in children with PH. Second, the different study design, disease type and cut-off of both BNP and NT-proBNP were not the cause of heterogeneity (*p* > 0.05); however, paediatric structural cardiac diseases, like atrial septal defects, ventricular septal defects, etc., could both cause PH and an increase in BNP/NT-proBNP, videlicet increased BNP/NT-proBNP might be related to the altered cardiac structures and not necessarily be related to PH. Therefore, the generalizability of the pooled diagnostic performance of BNP/NT-proBNP for paediatirc PH could be reduced. Besides, we did not evaluate other potential factors, like age, sex and body weight stratification, especially the published normal range levels of BNP and NT-proBNP showing age-dependent tendencies [43]. Third, even the different disease type (BDP-PH or not) was not the source of heterogeneity, further multi-centre and large sample studies could concentrate on some specific categories for paediatric PH, such as BPD-CH, CHD-PH, PPHN, etc. In addition, the severity of paediatric PH might vary, and it could affect the diagnostic capability of BNP and NT-proBNP. Fourth, wide CIs could be led by the small sample size, as the number of paediatric patients with PH per included study ranged from 5 to 65.

Furthermore, a relatively low risk of methodological quality bias was found in this study. One eligible study reported by Li et al. in 2016, showed the high risk of bias by the clear cut-off value of BNP [26]. No publication bias was found for both BNP and NT-proBNP. However, the number of eligible studies of BNP was less than 10, we could not ignore a concern for publication bias.

## Conclusion

This diagnostic meta-analysis shows that both BNP and NT-proBNP are good markers for differentiating paediatric PH patients from non-PH individuals. In clinical practice, we recommend that BNP and NT-proBNP are auxiliary biomarkers for heart catheterization or echocardiography in diagnosing paediatric PH. Further multi-centre and large sample studies are warranted to support our findings and determine whether these findings are correct.

## Supplementary Material

Supplemental Material

## Data Availability

Data are available of this study. All data are collected and analysed from the published studies which are served as references in this systematic review and meta-analysis. The corresponding author (Xia Qiu) could provide the raw data for statistical analysis by request.
