# Supplementary material for: Accuracy of brain natriuretic peptide and N-terminal brain natriuretic peptide for detecting paediatric pulmonary hypertension: a systematic review and meta-analysis
Source: Ann Med. 2024 May 16;56(1):2352603. doi: 10.1080/07853890.2024.2352603 (PMC11100439; doi:10.1080/07853890.2024.2352603)
Supplement: Supplemental Material [file IANN_A_2352603_SM9372.zip › suppl_data--2/Clean Supplementary_materials table.docx]

**Supplementary table 1.** Diagnostic performance of BNP and NT-proBNP in the included studies

| Author | Year | Samples | | Sensitivity (%) | Specificity (%) | TP | FP | FN | TN |
| --- | --- | --- | --- | --- | --- | --- | --- | --- | --- |
|  |  | PH patients | non-PH individuals |  |  |  |  |  |  |
| Jin N | 2023 | 65 | 92 | 86 | 85 | 56 | 14 | 9 | 78 |
| Nova R | 2022 | 33 | 42 | 55 | 81 | 18 | 8 | 15 | 34 |
| Tufekci S | 2022 | 33 | 50 | 91 | 96 | 30 | 2 | 3 | 48 |
| Zhang H | 2021 | 54 | 25 | 63 | 56 | 34 | 11 | 20 | 14 |
| Dasgupta S | 2021 | 10 | 21 | 60 | 90 | 6 | 2 | 4 | 19 |
| Griffiths M | 2020 | 26 | 21 | 76 | 81 | 20 | 4 | 6 | 17 |
| Naeem B | 2020 | 6 | 20 | 83 | 85 | 5 | 3 | 1 | 17 |
| Behere S | 2019 | 7 | 21 | 86 | 76 | 6 | 5 | 1 | 16 |
| Avitabile CM | 2019 | 68 | 60 | 50 | 92 | 34 | 5 | 34 | 55 |
| Rodriguez-Gonzalez M | 2019 | 21 | 72 | 86 | 89 | 18 | 8 | 3 | 64 |
| Dasgupta S | 2018 | 8 | 28 | 88 | 96 | 7 | 1 | 1 | 27 |
| Pektaş A | 2017 | 25 | 40 | 96 | 93 | 24 | 3 | 1 | 37 |
| Li G | 2017 | 46 | 39 | 85 | 67 | 39 | 13 | 7 | 26 |
| Li G | 2016 | 30 | 30 | 80 | 67 | 24 | 10 | 6 | 20 |
| Montgomery AM | 2016 | 5 | 15 | 100 | 93 | 5 | 1 | 0 | 14 |
| Cuna A | 2013 | 16 | 9 | 94 | 100 | 15 | 0 | 1 | 9 |
| Reynolds EW | 2004 | 15 | 15 | 80 | 100 | 12 | 0 | 3 | 15 |
| Suda P | 2003 | 30 | 29 | 83 | 90 | 25 | 3 | 5 | 26 |

Abbreviations: PH: pulmonary hypertension; BNP: brain natriuretic peptide; NT-proBNP: N-terminal pro-brain natriuretic peptide; TP: true-positive; FP: false-positive; FN: false-negative; TN: true-negative.
