# Supplementary material for: Accuracy of brain natriuretic peptide and N-terminal brain natriuretic peptide for detecting paediatric pulmonary hypertension: a systematic review and meta-analysis
Source: Ann Med. 2024 May 16;56(1):2352603. doi: 10.1080/07853890.2024.2352603 (PMC11100439; doi:10.1080/07853890.2024.2352603)
Supplement: Supplemental Material [file IANN_A_2352603_SM9372.zip › suppl_data--2/Supplementary_figure Caption.docx]

**Supplementary figure 1**. The pooled sensitivity and specificity of BNP/NT-proBNP for detecting PH in pediatric population.

**Supplementary figure 2a**. Deeks’ funnel plot asymmetry test of BNP/NT-proBNP.

**Supplementary figure 2b**. Deeks’ funnel plot asymmetry test of BNP.

**Supplementary figure 2c.** Deeks’ funnel plot asymmetry test of NT-proBNP.
