# Supplementary figures and images for: Accuracy of brain natriuretic peptide and N-terminal brain natriuretic peptide for detecting paediatric pulmonary hypertension: a systematic review and meta-analysis
Source: Ann Med. 2024 May 16;56(1):2352603. doi: 10.1080/07853890.2024.2352603 (PMC11100439; doi:10.1080/07853890.2024.2352603)

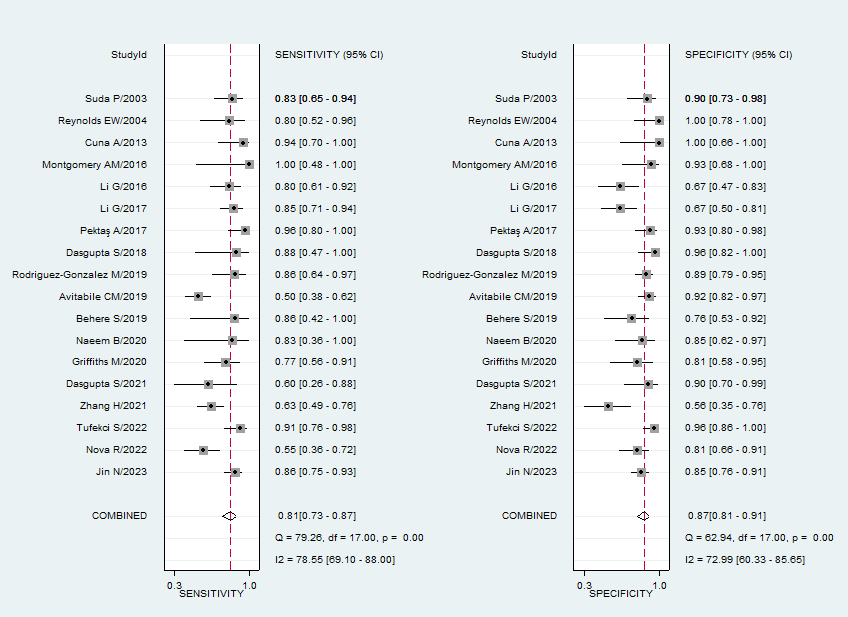

Supplement: Supplemental Material [file IANN_A_2352603_SM9372.zip › suppl_data--2/Supplementary_figure_1.tif]

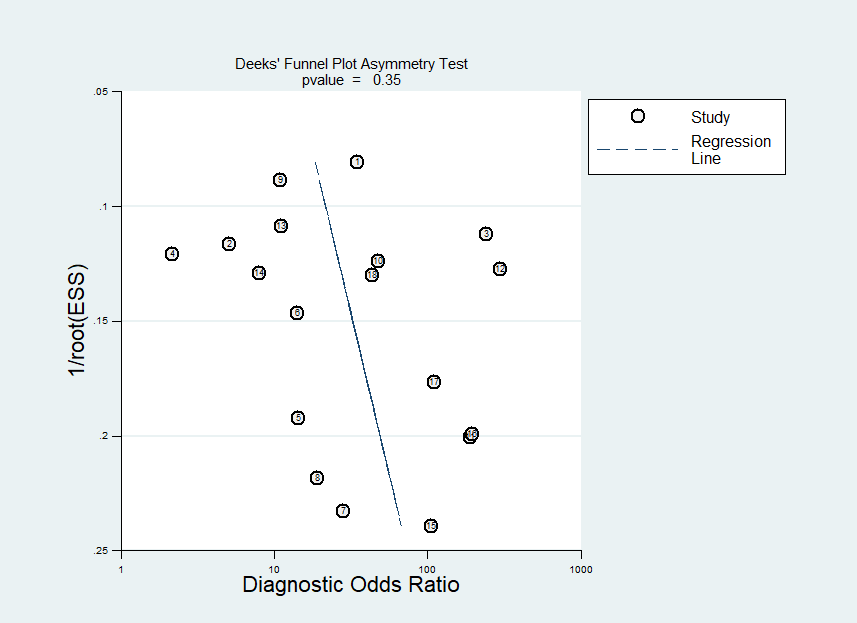

Supplement: Supplemental Material [file IANN_A_2352603_SM9372.zip › suppl_data--2/Supplementary_figure_2a.tif]

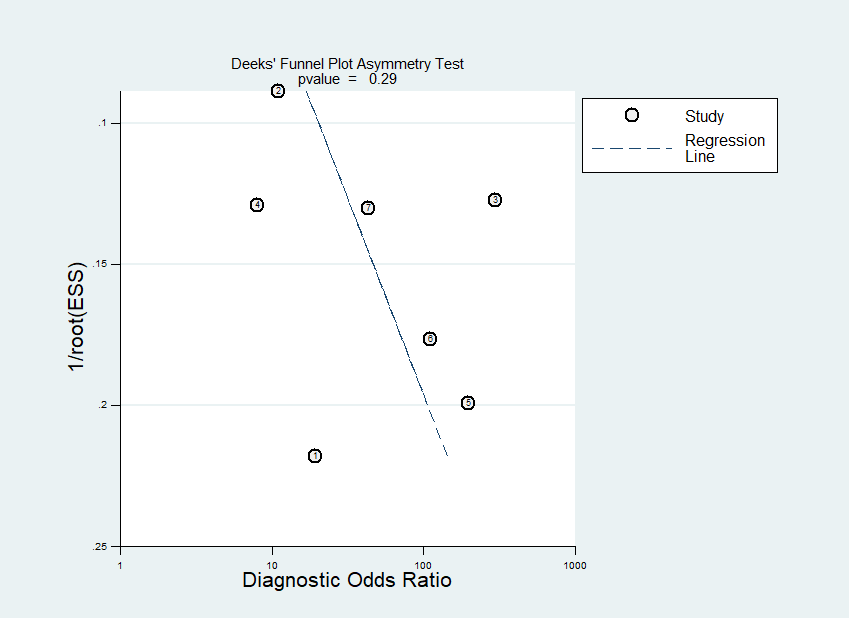

Supplement: Supplemental Material [file IANN_A_2352603_SM9372.zip › suppl_data--2/Supplementary_figure_2b.tif]

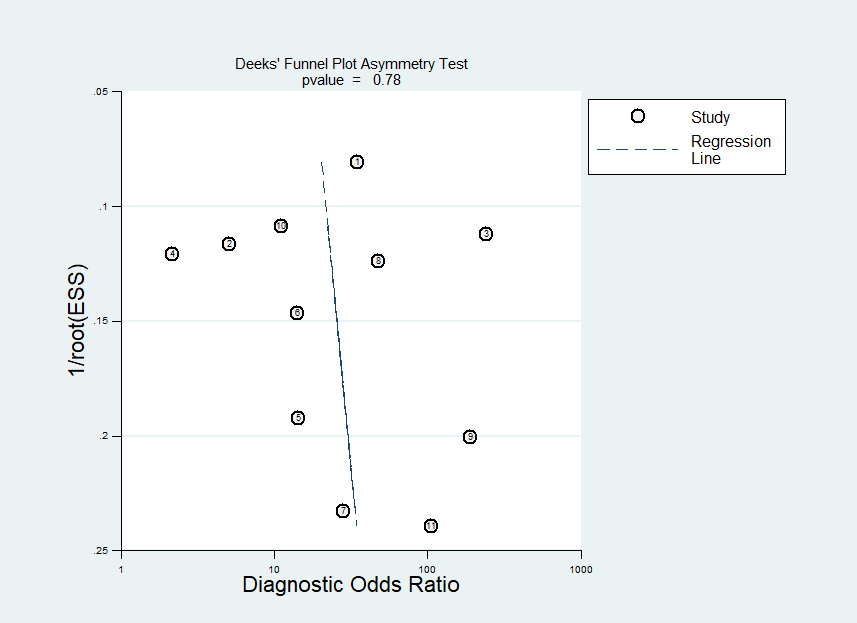

Supplement: Supplemental Material [file IANN_A_2352603_SM9372.zip › suppl_data--2/Supplementary_figure_2c.tif]
